# Very Cold Indeed: The Nanokelvin Physics of Bose-Einstein Condensation

**DOI:** 10.6028/jres.101.045

**Published:** 1996

**Authors:** Eric Cornell

**Affiliations:** JILA, National Institute of Standards and Technology and University of Colorado, Boulder, CO 80309

**Keywords:** atom laser, atom traps, Bose-Einstein condensation, evaporative cooling, superfluids and supergasses

## Abstract

As atoms get colder, they start to behave more like waves and less like particles. Cool a cloud of identical atoms so cold that the wave of each atom starts to overlap with the wave of its neighbor atom, and all of a sudden you wind up with a sort of quantum identity crisis known as Bose-Einstein condensation. How do we get something that cold? And what is the nature of the strange goop that results? These questions were addressed in a colloquium at the National Institute of Standards and Technology in Gaithersburg, Maryland, on February 23, 1996. This paper is an edited transcript of that presentation.

Today’s audience is a little bit large for this to be called a family talk, I guess, but it certainly is a treat for me to give a talk to a NIST audience.

The work that I am going to be telling you about today was done at JILA, a joint institute between NIST and the University of Colorado, and was done in very close collaboration with Carl Wieman, who is a University of Colorado professor and with whom I have been working now for 5 years.

As you can probably guess, Carl and I didn’t do very much of the actual work. A lot of that was done by a group of people at JILA, who are shown in Fig. 1.

We have in-house at JILA some very helpful theoretical support and have had distinguished visitors over the years, and also have various sources of in-house and out-of-house funding.

Let me describe what Bose-Einstein condensation is and particularly why it is hard to get there, tell you how we were able to do it, and then outline what it is that we want to do with these condensates. This is also a way of explaining why it is we wanted to make them in the first place.

Everything in the world, certainly all particles, but even composite particles and cars and everything else can be divided into two big classes, called bosons and fermions.

Bosons are particles which have integer spin; the angular momentum of the particles is 0, 1, 2, 3, and so on, in units of the reduced Planck constant *ħ* = *h*/(2π). Fermions are particles which have half-integer spin: 1/2, 3/2, 5/2, and so on, in the same units.

There are some classic examples. Both photons and phonons are examples of bosons. A large proportion of the atoms that you see around you are also bosons, rubidium-87 atoms (^87^Rb), for example, just to telegraph where we are going.

Fermions are, if anything, even more common. Most of the elementary building blocks of fundamental stuff are fermions: electrons, neutrons, protons, the things inside neutrons and protons. A smattering of atoms, including helium-three (^3^He), are also fermions.

Of course, all atoms are made up of a bunch of fermions stuck together. But if you stick together an even number of fermions, you get a composite particle with integer spin, which is a boson.

Why do we care about it? Well, for mysterious reasons, the spin of a particle has a lot to do, if we can anthropomorphize a little bit, with defining its personality.

Bosons like to be in the same state. Bosons are sociable. They are gregarious. Fermions, on the other hand, are loners. To be a little bit more technical about it, this antisocial behavior of fermions is what gives rise to the Pauli exclusion principle.

That is why you can only have one electron in each orbital around an atom; you cannot have more than one fermion doing the same thing.

Bosons, on the other hand, love to all do the same thing. For instance, in this laser pointer which I hold in my hand, there are a tremendous number of photons first bouncing back and forth inside it and then spilling out one end. They are all going exactly the same direction with the same energy.

They are actually in a form of condensate themselves coming out here. So that’s a multimedia demonstration of the gregarious nature of bosons.

It was Bose who suggested that this gregarious behavior in photons could actually account for something which happens when an object gets very hot, which is black body radiation.

Einstein was very excited about this, and in 1925 he pointed out that you could apply some of the same rules to other bosonic particles and derive a concept known as the Bose-Einstein distribution.

Basically, you can think of how he got the Bose-Einstein distribution by playing a statistical mechanical game. Start off with a box. The box contains particles, and its confinement gives rise to a bunch of quantum states, which are just allowed places where you can put the particles in.

Take a fixed number of atoms that share among themselves a fixed amount of energy. The statistical mechanical rule allows you to put any number of these indistinguishable bosons into any given state.

Now you play a game, which is to distribute the particles among these states in a way which is maximally random while still following the statistical-mechanical rule. The most random distribution is called the Bose-Einstein distribution. A picture of it is shown in Fig. 2.

This is the average number of particles in level *i*. The energy of the level is *ϵ_i_*. This *μ* and *T*, if you are feeling mathematical you can think of them as Lagrange multipliers, but more familiarly they are known as the chemical potential and the temperature. Basically you sort of pick these in order to get your energy and number right. This shows you how you spread the particle distributions around.

This function looks a lot like the more familiar Maxwell-Boltzmann distribution, if you ignore the −1 in the denominator. But you cannot ignore the −1: it is what gives you all the action in this distribution.

So in the game you pile the particles into the box. Most of the particles go down into the low-energy states, and then there is a tail in the distribution at high energy. This is pretty standard thermal physics. As the system is cooled down a little bit, the particles pile up a little bit more in the low-energy states.

But when you get sufficiently cold, something remarkable happens very suddenly. You get a tremendous number of particles all sitting in the very lowest available energy state in the box in which you are holding the atoms. This causes a spike to appear in the energy distribution at the origin: it is the formation of the Bose condensate.

I will not derive the expression for the conditions in which this transition occurs. It describes a particular point in phase space, at which the product of the coldness and density of the atomic gas gets sufficiently high, see Fig. 3.

We know that photons have a wavelength. It turns out that atoms also have a wavelength, and it depends on their momentum. The basic idea is that particles are a little bit like waves, waves are a little bit like particles.

As the atoms get colder and colder, their wavelength, which is called the de Broglie wavelength, gets longer and longer; the fuzziness, if you like, of the particle becomes more and more pronounced.

In a gas of identical boson atoms, when the particles get close enough together, or as they become so cold that the de Broglie wavelength of one atom overlaps that of another, the atoms have a sort of quantum identity crisis. At that point Bose-Einstein condensation kicks in. This occurs at a phase space density of about one, in the natural units of the Planck constant, *h*.

In the approach to condensation, if you look at the number of particles in the lowest energy state, which is the nodeless wavefunction at the very bottom of the box, you will find that there is only one or maybe a few atoms in that state.

Then, as the gas is cooled to the transition point, the number of atoms piled up there at the very bottom suddenly goes up through the roof.

If the number of particles in the system is comparable to Avogadro’s number, *N*_A_ = 6 × 10^−3^ atoms, which is characteristic of macroscopic objects, then even if you’re just a very few percent below the critical temperature you have a significant fraction of Avogadro’s number of particles all doing the same thing.

A common misconception about Bose-Einstein condensation is that it requires brute force cooling. The mean energy of the particles is given by the product *kT* of Boltzmann’s constant and the absolute temperature. So if you were to get the temperature very, very low, it would be no surprise that all the particles were in the lowest energy state, because they would not have energy enough to go anywhere else. But condensation can happen at much higher temperatures, when *kT* is still large compared to the differences of energies of the quantum states of the system. That is a note for the experts.

The classic example of Bose-Einstein condensation for many years was liquid helium. At the transition of liquid helium from an ordinary liquid to what is called a superfluid, the viscosity vanishes and helium starts to behave like a quantum fluid. The phase-space density at the transition point is right at the number that you would expect it to be if helium were in fact a Bose-Einstein condensate.

Most people believe that helium is a sort of Bose-Einstein condensate. But it is a liquid and not a gas, and the helium atoms in the liquid interact quite strongly. The system is difficult to understand on an elementary level. So there has been a push for many years to try and see Bose-Einstein condensation in something closer to a gas.

Why has it taken so long? Why is Bose-Einstein condensation hard? Here is a general descriptive picture of the problem, portrayed in phase space (Fig. 4). It is basically applicable to all substances.

The vertical axis labels the temperature of the system, the horizontal axis its density. The green line is a phase boundary. The exact location of that green line can move around a little, but it will be present for just about any substance. At low densities and high temperatures, everything is a vapor. At high densities and lower temperatures, everything is condensed, into either liquid or solid form.

Underneath the green line there is a huge area that you cannot get to in conditions of thermal equilibrium. It is called the forbidden region.

For instance, if you were to take a box, hold it at a certain temperature and put enough atoms in it to drive the mean density into the forbidden region, you still would not find any stuff with this density and temperature. It would separate out.

At the bottom of the box, there would be a high-density lump of stuff, like water or ice; at the top of your box, there would be vapor; and there would be nothing in the forbidden region.

Why does this matter? It matters because the transition to the Bose-Einstein condensed phase, which is shown as a blue line in this figure, is always deep down in the forbidden region. The relative locations of the green line and the blue line for any material are just as depicted here, except for liquid helium. So liquid helium is the only substance that can be Bose-Einstein condensed under normal thermal equilibrium conditions. Everything else solidifies in that range of density and temperature.

You have all seen pictures produced by scanning tunneling microscopes where the each individual atom can be seen. Well, if you can see each individual atom, they are not really indistinguishable anymore, so the rules of Bose-Einstein distribution are off. You cannot get a solid to form a Bose-Einstein condensate.

Below the blue line is where we want to go. We can see that this line lies in the forbidden region. Is it impossible to go into this forbidden region? To paraphrase an old joke by Joseph Heller, if it were truly impossible, they would not have bothered to forbid it. This is encouraging!

How do you get into the forbidden region? You use a fairly familiar phenomenon called metastability.

Say you have some gas in a box in thermal equilibrium and it’s kind of cold, say around 200 K. The vapor will be neither cold enough nor dense enough to get to Bose-Einstein condensation. The way we get it to condense is to start off with a thick vapor at a high temperature and to cool it down very slowly. If it is cooled very slowly, we can wind up with a thick vapor at low temperature without a condensed phase ever forming.

The reason is that ice or crystals or droplets need something to nucleate around. You are probably familiar with this. If you have a gas, you can actually cool the gas down below the temperature at which it liquefies because there are no places for the droplets to form.

When the droplets do form, they usually form on the walls or on some dust or other impurities in the system. If you have a very clean system with no dust, and if you can somehow prevent your atoms from touching the walls, you may be able to go deep down into the forbidden region without forming the condensed phase. You enter the forbidden region in a metastable state, so called because it remains stable if there are no nucleation centers.

All Bose-Einstein condensation efforts to date have been attempts to reach this metastable state before the atoms realize that what they really want to do is to form into thick ice in a thin vapor. The trick is to remain at very low densities, so that three-body atomic collisions are unlikely. Even if atoms don’t touch the walls, three-body collisions can cause them spontaneously to start forming molecules, and the molecules can spontaneously form into droplets or clusters. Two atoms by themselves can’t form a molecule because they come together and bump away, and there is nothing to cause them to stick together. But if three atoms come together at the same time, two of them can collide and stick together as a molecule, and the third atom can take away the extra energy. Once you have molecules, they very quickly accumulate into snowflakes.

So as long as you can keep very low densities so that the atoms can’t take that first step and form a molecule, you’ll be okay.

Let’s have a look at efforts to see Bose-Einstein condensation. Some of the people who really pioneered many of the ideas in this field are those who worked with spin-polarized hydrogen atoms: for instance, Walraven at Amsterdam, Silvera at Harvard, Kleppner and Greytak at MIT, and the group at Cornell. They took hydrogen molecules, dissociated them into atoms, and put the gas in a dilution refrigerator at a particular temperature. And they just pressed it closer and closer together. But before they could get to the transition line, the atoms got so dense that they started to form molecules via the three-body recombination mechanism I’ve just described.

Other trappers took atomic hydrogen, and instead of compressing it, they cooled it using a technique called evaporative cooling. They also almost got to Bose-Einstein condensation. This encouraged us to give it a shot using a similar technique. We realized we had to get down to very low densities so that we don’t have three body recombination. If you go to low densities, you have to go to very low temperatures. That’s why we’re stuck with these extraordinarily low temperatures to get to Bose-Einstein condensation.

An experiment which is not widely known but which I think is quite beautiful is work done on excitons, which are bound pairs of electrons and holes in certain glasses. The exciton gas can be cooled to get Bose-Einstein condensation. This is Jim Wolfe’s work at the University of Illinois.

Just to give you an idea of the scale and also to give away the punch line, we were able to do this in rubidium recently at a very low density: ten orders of magnitude lower than the density of liquid helium. Not surprisingly, we also had to be nearly ten orders of magnitude colder than liquid helium to get to Bose-Einstein condensation.

How do we possibly get anything this cold? First, we used laser cooling of alkali atoms. This is a technique which was largely pioneered here at NIST, but also at Bell Labs, at the Ecole Normale in Paris, at JILA, and at MIT.

Now we will have a quick review of laser cooling. It is an extraordinarily rich topic which deserves a colloquium in and of itself. But what I am going to tell you instead is the encapsulated version.

Laser beams, in addition to carrying heat, also carry momentum. If you go out and stand in the sunshine, the light hitting you on one side applies a very small pressure to you. The force from this light is actually very small. On the other hand, the mass of atoms is also very small. When you divide something small by something very small, such as the light force on an atom by the atomic mass, it turns out in this case that you get something kind of big. The acceleration of an atom due to the light force can be 10 000 times the acceleration of gravity. These are big forces from an atom’s point of view.

But you need more than just a force. You need somehow to apply these forces on atoms to get them to slow down, i.e., make them colder. The way laser cooling works is to bring in the laser beams from two directions, say, one from the left and one from the right. Now suppose I’m an atom moving to the right. To slow down, I have to absorb a photon coming toward me (to the left), and not one coming from behind me. This can be arranged by use of the Doppler shift.

Here’s how it works (see Fig. 5). This is a resonant curve showing the atomic frequency response, i.e., how much the atoms are likely to scatter photons as a function of the laser frequency. In Boulder, most of the year it’s snow peaked at the top.

We tune the laser frequency a little bit to the low-frequency (“red”) side of the resonance. The laser beam opposing the atom is Doppler shifted to a higher (more “blue”) frequency. Thus the atom is more likely to absorb that photon. A photon coming from behind the atom is now a little bit redder, which means the atom is less likely to absorb that photon. So in whichever direction the atom is moving, the laser beam opposing the motion seems stronger to the atom, and it slows the atom down.

If you multiply this by three and have laser beams coming in north, south, east, west, up, and down, you get what’s called optical molasses. If you walk around in a pot full of molasses, whichever direction you go, the molasses somehow knows that is the direction to push against. It’s the same idea.

You can get atoms very cold in this way. We do all our experiments in a little glass box. The laser beams push the atoms into the middle of this little glass box and cool them. The atoms get very cold, to temperatures of about 10 μK and densities of 10^11^ atoms per cubic centimeter, which is a phase space density of 10^−5^ in the natural units. The density required for Bose-Einstein condensation is about one in these units. So even though the atoms are extraordinarily cold and rather dense for an ultracold vapor, they’re still a long ways away from Bose-Einstein condensation.

What keeps them from getting any colder, by the way, is basically Brownian motion. Say you have a grain of smoke moving along: the air seems viscous to it and it quickly slows down. The air is acting like molasses to the little grain of smoke. But it doesn’t come to a complete stop. In this situation, if you look at the smoke grain under a microscope, it will be jittering around.

Why does it jitter around? It does so because of the discrete nature of air. Random lumps of air (molecules) are hitting it from one direction or another.

Light, as we know, also comes in little lumps called photons, and this gives rise to an effect analogous to the little jitter of Brownian motion. The final minimum velocity we can obtain in optical molasses is completely analogous to the little jitter of the smoke which you sometimes see when it comes to a stop in air.

Laser cooling is thus a first step. It gets us more than half way to Bose-Einstein condensation. Now we need a new kind of cooling to go beyond that. Happily, people like Bill Phillips’ group here for instance, have provided us with a different way of holding atoms.

We turn the lasers off! The first inclination of the atoms is then to fall under the influence of ordinary gravity. If there are no forces on them, they just plain fall, and, if they touch the walls of the box, they will solidify there. We need to hold the atoms up. Fortunately, they are not moving very fast: by the time they have undergone laser cooling in the optical molasses, they are only moving a few centimeters per second. So they can be confined by a relatively weak magnetic field (see Fig. 6).

Each alkali atom, rubidium in our case, has got an unpaired electron with a magnetic moment, which is in the direction opposite to the electron spin. That magnetic moment interacts with a magnetic field, so we can use magnetic fields to push the atoms around.

In fact, if the electron’s magnetic moment is parallel to the magnetic field, it’s attracted to a local minimum value of the magnetic field strength, and just sits there.

We can also arrange for it to be repelled from the minimum field position by reversing the direction of the magnetic moment. But if the electron spin is pointing the right way, magnetic fields can be arranged to form a little bowl, and atoms will be trapped in this little bowl.

This bowl is terrific because it can be used for a kind of cooling called “evaporative cooling.” This kind of cooling was pioneered by the spin-polarized hydrogen trappers at MIT. It’s really a terrific idea. It’s the hottest thing in cold!

But in some sense, it’s also one of the oldest cooling techniques around. Any time you get a cup of coffee, it cools evaporatively. If you get a cup of coffee to go, and you don’t keep the lid on it, it cools quite quickly. That steam you see coming up is there because of evaporative cooling (see Fig. 7).

The way to think of it is that the cup contains a large collection of coffee molecules with a variety of energies.

A few of the coffee molecules have enough energy to break out of what is known, technically, as the coffee work function. They break out of this little energy barrier at the surface.

The only molecules that can evaporate are those which have much more energy than the average coffee molecule. So when they do get out, the molecules which are left behind have a lower average energy. The coffee gets cooler.

This is something we are all familiar with. But you may never have stopped to think about what a really terrific method of cooling it is, and how efficient it is.

If you buy a cup of coffee to go, it comes in a insulating styrofoam cup; so most of the heat comes out from the steam. The coffee has a temperature of close to between 370 K and 373 K.

If you don’t put the lid on it, in a relatively short time scale, it cools down to about 300 K. So the temperature has changed by about 20 %.

Yet if you let that coffee just sit on your desk as it cools down, you will see a little stain appear on the inside of the cup. That stain tells you how many coffee molecules have escaped due to evaporation; it works out to be only 2 % of the coffee molecules in the cup.

Think about it. You lose 2 % of the molecules in your cup, yet you lower the temperature by 20 %. Suppose you could lose another 2 % and lower another 20 %, and so on?

This shows that the temperature of the coffee scales as the tenth power of the number of atoms remaining in the coffee cup. That’s a very strong power. In fact as we all know, if you do not keep the lid on your coffee cup, by the time you get home it’s quickly cooled to absolute zero!

Well, it doesn’t actually cool to absolute zero. Perhaps you have thought to wonder why. Go out in the desert somewhere where the molar concentration of coffee in the atmosphere is zero. Yet still the coffee doesn’t get that cold; it stops after awhile.

The reason for this is that after the coffee is sufficiently cooled, there are not enough atoms left with enough energy to break out of the cup. The evaporative cooling rate has basically vanished.

In our experiments, the same thing is happening. We put the atoms into a bowl and take the lid off, so to speak. We put a little distortion, a lip, in the bowl. Then only the atoms that have extra energy can come out over the lip. The remaining atoms have a lower average energy, and they cool down (see Fig. 8).

The great thing, though, is that as the atoms cool down, the trap can be continuously distorted, so that even when the atoms are cold, the height of the lip with respect to the average energy of the atoms is still just a few times the mean energy.

In effect, we are artificially reducing the work function of our cloud so atoms can continually fall out.

As the atoms cool down, they occupy a smaller and smaller volume because they don’t have enough energy to roll so far up the side of the bowl. Thus, their density *increases* even as their temperature goes down.

This is exactly what we want to happen to get Bose-Einstein condensation.

The trap we use has a pointy bottom. When we jiggle the bottom around we get this nice rounded confining potential, which is parabolic. We call this a TOP trap: TOP stands for “time-averaged orbiting potential.” It spins around like a top actually, and that’s why we call it TOP. Also by “top” we mean that it’s a really great trap.

Now I’ll give you a summary of the whole experiment. First, we collect the atoms using laser beams and a magneto-optic trap (MOT). Then we cool them down in the optical molasses.

We do some optical pumping which puts all the atoms into the same spin state so they’re all lined up with the magnetic field and attracted to the local minimum.

Then, very suddenly, we turn on the magnetic trap and start evaporative cooling. We let the atoms out by applying a radio-frequency (RF) oscillating magnetic field which drives transitions in the atoms between the spin “up” state, which is attracted to the magnetic field minimum, into the spin “down” state, which is repelled from the trap.

Think of one of the hotter atoms. It rolls up high on the side of the bowl, comes into resonance with the RF spin-flip transition, and then falls out of the bowl. By gradually turning down the frequency of the RF magnetic field, we move the effective location of the lip of the bowl inward toward the center.

The frequency of the RF magnetic field is the experimental control parameter. We gradually turn down the frequency, slicing deeper and deeper into the atomic cloud, cooling the atoms that remain until we reach very low temperatures.

Figure 9 shows what the apparatus looks like. People are disappointed when they come to our laboratory. Because we have the world’s lowest temperatures, they wonder, where is the liquid nitrogen? Where is the liquid helium? Where are the vapors boiling off?

In fact, everything in the experiment is at room temperature, about 300 K, except for the atoms which are at about 300 nK. Everything happens inside the glass chamber in the center of the figure. The glass chamber is 2 1/2 centimeters across.

The coils above and below the glass chamber generate the magnetic field. Some additional coils and other folderol have been removed from this picture so that you can see the chamber itself.

The only other thing in this picture which really matters is the lens. You can think of this lens as the objective lens of a microscope we use for looking at the atoms.

Looking at the atoms with light gives us all the information that we ever extract about them. We can’t go in and touch them with a conventional thermometer, because such a thermometer would be tremendously hotter than the gas and would boil it away.

Figure 10 shows how we take a picture of the atoms. A laser beam, tuned to resonance with an atomic transition, is sent through the atomic cloud. The atoms scatter this light in all directions. Thus, the laser beam that comes through has got a shadow on it. We image that shadow onto a charge-coupled device (CCD) array. The dark areas correspond to regions of high column density of the atoms, i.e., we have a large value of the atomic density integrated along the line of sight. The edge of the image, where the shadow is not very dark, corresponds to the edge of the atomic cloud—the integrated density of atoms along that line is smaller.

We extract all the relevant thermodynamic information from those pictures in the following way. Since the shape of the potential is parabolic, we know how strongly the potential holds the atoms. Since the cloud is an ideal gas, its size tells us how hot the atoms are. We measure the size of the cloud and determine its temperature. Then we induce some evaporation. We turn on what we call the RF “scalpel.” This is the RF magnetic field I mentioned earlier. We slice away at the edge of the cloud, and then go deeper and deeper into the cloud. If this is done slowly enough, the cloud actually shrinks away from the scalpel. Only the highest energy atoms fall out, and the cloud gets much smaller. For instance, if a cloud gets ten times smaller in a linear dimension, it must become about 100 times colder.

The size of the shadow thus gives useful thermodynamic information about the cloud, and so does the darkness of the shadow. If the cloud gets ten times smaller in a linear dimension, but its shadow has the same darkness, this means the atomic density must have increased by a factor of ten. Since it is also a hundred times colder, we have obtained an increase of four orders of magnitude in phase space density. These are very typical numbers for our system. I should mention that similar increases due to evaporative cooling have been reported by Randy Hulet’s group at Rice University and by Wolfgang Ketterle’s group at MIT, and the MIT group has indeed recently demonstrated Bose-Einstein condensation in an evaporatively-cooled gas of sodium atoms.

Our evaporated cloud is now very close to attaining Bose-Einstein condensation. Unfortunately, the cloud is also quite small, near the limit of the spatial resolution of our imaging system. So we decided to make the cloud bigger before taking its picture.

We do that by first slowly reducing the spring constants in the parabolic trap and then, quite suddenly, turning the trap off. The atoms just find themselves in free space. What do the atoms do then? They have some residual velocity, so they just fly apart. After they have flown apart for a time, the cloud is much bigger, and we can take its picture.

Since we know how long the atoms were flying apart, we can do a time-of-flight measurement to determine their speeds. The atoms on the outside of the cloud must have been going very fast when we released them, and the atoms near the middle of the picture must have been essentially stationary. So this picture shows the velocity distribution of atoms in the cloud at the time of its release, instead of the spatial distribution. It turns out that there is not much qualitative difference between the two, for in a parabolic trap such as ours, the spatial and velocity distributions are proportional.

Now let us ask what we expect to see. Figure 11 is an artist’s conception of Bose-Einstein condensation. Before we cool below the critical temperature, the atoms are thermally distributed among all these different levels. If you take a picture of them, they look like a big fuzzy ball. Below the critical temperature we have a large number of atoms all occupying this lowest energy state. In our harmonic oscillator, that lowest energy state is highly localized in both coordinate space and in velocity space. The atoms on the bottom are not moving very much, and are all clumped together. So if we take a picture of the cloud, we see a dense spot in the middle. A cross section of the density distribution would show a two-peaked profile: a big, broad cloud of thermal atoms, and a sharply-peaked condensate cloud. We are looking for such a profile as we cool the system down, hoping to see a sudden increase in the density right in the center of the cloud.

This is depicted in Fig. 12, which shows the central density vs the frequency of the RF scalpel. As the frequency decreases, we are cutting deeper and deeper into the cloud. You can see that the density of the cloud increases slowly as we cool it down, which is just a consequence of the normal process of evaporation.

Then, quite suddenly, we come to some sort of critical point and, wham, the density just goes through the roof. This is the onset of Bose-Einstein condensation! (It is interesting to note that if we keep cutting deeper and deeper, the density comes back down again, because the scalpel starts to cut into the condensate itself.) Figure 13 shows three clouds getting progressively colder as we cut deeper. The first cloud is a thermal cloud; you can see that it is round, smooth, and bigger than the others. When we cool this cloud down a little bit, a spike appears right in its middle. It corresponds to atoms which are hardly moving at all. That is the condensate appearing. You can see that we have a two-component cloud. The superfluid helium people would see this as the presence of a normal liquid and a superliquid.

If we continue to cool even further, we can shave away the normal cloud, and are left with only a pure condensate. The rightmost frame of Fig. 13 is macroscopic in size; you can just see it with your bare eyes if you squint. The long dimension of this cloud is about 40 μm to 50 μm. So it is much bigger than a wavelength of light. This picture is thus an actual photograph of a single macroscopically occupied wave function. There are 1000 or 2000 atoms participating in this wave function, all doing the same thing.

There are a couple of interesting things we can see about this cloud right away. For instance, notice that the thermal cloud is round, whereas the condensate cloud is elliptical. Figure 14 shows this same data looking down from above, and again, the thermal cloud is round, but the condensate is elliptical. The roundness of the thermal cloud indicates that the velocity distribution of its atoms is isotropic, i.e., the same in all directions. This is just what basic statistical mechanics tells us it should be: the equipartition theorem states that no matter what the shape of the trap, the velocity distribution has to be isotropic. In fact, that the potential in which we hold the atoms is not spherically symmetric, but cylindrically symmetric. The atoms are squeezed more tightly along the trap symmetry axis (the axial coordinate) and less tightly in the direction perpendicular to that axis (the radial coordinate).

In contrast to that of the thermal cloud, the condensate’s velocity distribution is elliptical, not spherical. What’s going on? It can be explained by Heisenberg’s uncertainty principle. Atoms in the condensate are not thermal objects at all. The condensate must be consider in the very bottom of the trap. The condensate is smaller along the axial direction than the radial direction because the axial forces in the trap are stronger. What does the uncertainty principle tell us about this? It says that if you know really well where a quantum object is, you can’t really know how fast it’s going; and on the other hand, if you know less well where the object is, you can have a better idea of how fast it’s going. If the object is bunched up in coordinate space, it will be spread out in momentum space, and vice versa. We actually get a demonstration of the uncertainty principle at work when we turn off the trap, let the atoms fly apart, and take a picture of their momentum distribution. Sure enough, the cloud, which was initially squeezed up in the axial direction in coordinate space, is now more spread out in that direction. So this is quantum mechanics at large.

Okay. Wrapping up. I have not mentioned a whole lot of numbers because they’re so small that they don’t mean a lot. But for those who are interested, as we go through the transition the temperature is about 100 nK and the densities are about 5 × 10^12^ atoms per cm^3^. This is extremely rarefied by any normal standard. This is 10 orders of magnitude lower than the density of stuff in condensed matter, and many orders of magnitude lower than even the air in front of you. So it’s a very rarefied cloud. The only reason we can see it so well is that the atoms scatter light very strongly.

After we go below the transition, all the atoms pile up in the middle. The density gets much higher. The temperature becomes hard to define but, if you were to call it anything, it would be just about zero, because you have all the atoms in the single wave function; there is not a lot of entropy there. It’s a very low temperature. When the atoms are flying apart, you can talk about their mean kinetic energy, which used to be related to temperature. We can at least call it a temperature. Its value can now go below one nanokelvin. So to borrow Bill Gadzuk’s phrase, by getting down to 500 pK we’re doing picoKelvin physics—or at least half-nanoKelvin physics. Okay, what comes next?

The properties of the Bose condensate are largely unexplored. When I came into NIST this morning I saw a poster for my talk, and I saw to my horror, that in my abstract I promised to sort of explain how it all worked and what it all meant. And I thought maybe I was too optimistic when I wrote the abstract.

There are some things which are very clear. You have a whole lot of atoms. They’re all doing the exactly the same thing. They’re all participating in the same wave function. They’re overlapping. They are not necessarily very close together in the classic sense of many atoms occupying a small volume. In fact, the average density of our condensate is very low—one billionth the density of normal solids or liquids. But the atoms in the condensate are coherent, whatever that means.

Let’s talk about some experiments that we will be able to do. One of the neat things about many of these experiments is that we don’t really know what’s going to happen, which makes them particularly exciting.

First of all, I should mention that there are two familiar topics in physics that are related to Bose-Einstein condensation. One of them is superfluid liquid helium, helium which is so cold that the viscosity has entirely vanished. The other is the laser. We don’t often think of a laser in terms of a Bose-Einstein condensate, but here is a way of seeing the analogy.

Think of the laser in terms of a cavity with mirrors on each end, that contains a large number of normal modes of the electromagnetic field. When the lasing condition is established, we have a whole bunch of photons all in the same mode. It’s more or less like a Bose-Einstein condensate of photons.

Now we can draw on the superfluid and laser analogies to identify two new families of experiments (see Fig. 15). First, we can do experiments analogous to the classic studies of very low-temperature fluids. We can look at the change in specific heat associated with Bose-Einstein condensation; in liquid helium, for instance, the change in specific heat exhibits strange behavior called the lambda transition. We can look at excitations, which amount to sound waves in the linear regime. The first sound, something called second and even third sound exists. There may be analogous phenomena in our condensate. We can look at stronger, collective excitations, such as vortices. I should mention that a lot of the theory for this is now being done here in Gaithersburg. Vortices are well-known in liquid helium. If you spin a container of liquid helium, little dimples form in the fluid, and they persist even after the container stops spinning. The helium sitting in those little dimples keeps spinning forever. It’s an amazing phenomenon. If you were to unplug your bathtub and start a little whirling water, and then put the plug back in and see the water keep whirling, you’d call that very weird. Well, we think it will probably happen in Bose-Einstein condensate, if we could get the thing spinning. We should be able to form persistent currents in this substance. Let’s call it a supergas, in analogy with superfluids and superconductors, which display persistent currents of other particles.

By the way, I’ve never been very comfortable with the idea that our Bose-Einstein condensate represents a “new phase of matter,” and I’d like to dissociate myself from it a bit. For most purposes, you can think of the condensate as a gas. It has the same sort of correlations between the particles; it acts gas-like to a physicist. In the same way, a laser does not represent a new form of energy; it’s just light. So you wouldn’t call a laser a new form of energy, you’d call it coherent light. The Bose-Einstein condensate is coherent gas, or, following our analogy, a supergas.

Now I want to emphasize that by looking for persistent currents and the like, we are not just redoing the superfluid liquid helium experiments, because we obviously have a much different system. We also have a new set of probes, which in many ways are more powerful. As we know, it is possible to see individual atoms. We can resolve the atomic velocity distributions, something which has not really been possible to do in liquid helium. There are also things we can’t do. We can’t stick a little paddle in the condensate and swirl it around. But that’s okay—you lose a little, you win a little.

One of the biggest differences between the supergas and superfluid/superconductor systems is in the underlying theory. In our supergas, the cloud is so thin that you can actually calculate what’s going to happen in the regime known as perturbation theory, for the experts out there. The atoms are far enough apart that their interactions are just a perturbation of the ideal gas. You can calculate the condensate behavior and understand it without doing large-scale computations. So if I wanted to get grandiose, I’d say this unifies our heuristic, and our formalistic understandings of the quantum mechanics of many-body systems.

Now let’s go over to the laser picture. Let me first pose the question—how does a Bose-Einstein condensation form? It’s very analogous to lasing. In a laser, we have a collection of some sort of excited material that is putting out photons. Before lasing action occurs, the material is throwing off photons in all directions. If we put mirrors around it, some photons that go out hit the mirrors and come back. Pretty soon you get photons which are all doing the same thing, and when new photons come out of the active material, they prefer to go into the modes which are already occupied. The probability of a photon going into a mode is proportional to the number of photons already in that mode. This is because photons are bosons, as are rubidium atoms.

So now we can see the analogy of Bose-Einstein condensation with lasing. When two rubidium atoms collide with each other, they go off in new directions. They would prefer, if they had the option, for one of the atoms to go off into a mode of the atomic trap which is already occupied by other atoms, beccause, as in the case of the laser, that probability is enhanced in proportion to the number of atoms that are already in the mode. So the way that the condensate forms is that we get a little fluctuation, which puts a few atoms in the bottom of the trap. Then it’s much more likely for more atoms to fall in there, and we get a runaway process like that of the laser.

There are many ways of pushing the laser analogy further. Particularly here at NIST, people are already doing really interesting things with atom optics. If you have a Bose-Einstein condensate, you can now start to do interesting things with atom quantum optics, such as looking at correlations in arrival times. And let’s not forget that what a laser is best at is being a very nice source of photons. It’s very bright, since all photons are going in the same direction. This is the same in a Bose-Einstein condensate of atoms: the atoms all have the same energy. If we turn off the trap, the atoms would all fall down in an ideally monochromatic beam. We could use such a beam for studies analogous to those of photon correlations in quantum optics. We could perhaps use it to produce entangled states, which are of particular interest in understanding quantum measurements. The condensate might find applications in precision metrology. If you do a resonance experiment on a Bose-condensed gas, the inhomogeneous broadening vanishes completely. We won’t get into the systematics here, but for the precision metrologists in the audience, it’s something to think about: the broadening is identically zero. Thus we can see very narrow resonances.

The most straightforward way of doing such an experiment is to drop the condensate and make observations in the falling cloud. The measurement would last as long as the time it takes the cloud to pass the field of view of the apparatus. Over the course of that period, we’d say that pulse of atoms was transform-limited; the spread of energy is limited only by the observation.

If we can think of a way to take the atoms out of the trap little by little, we could get a continuous beam of atoms with a much longer coherence length, a much smaller spread in energy than we can obtain in the transform limit. People call this possibility the “atom laser.” In a laser you can have light coming out that’s organized, that’s coherent on a length scale which is much longer than the size of the laser cavity. Some people call the atom laser a “Boser.” I didn’t invent that terminology.

Anyway, to summarize these prospects in the deep voice version, we are going to enter a new regime of precision in atomic manipulation and in atom-based metrology.

Thank you very much for your attention.

## Figures and Tables

**Fig. 1:**
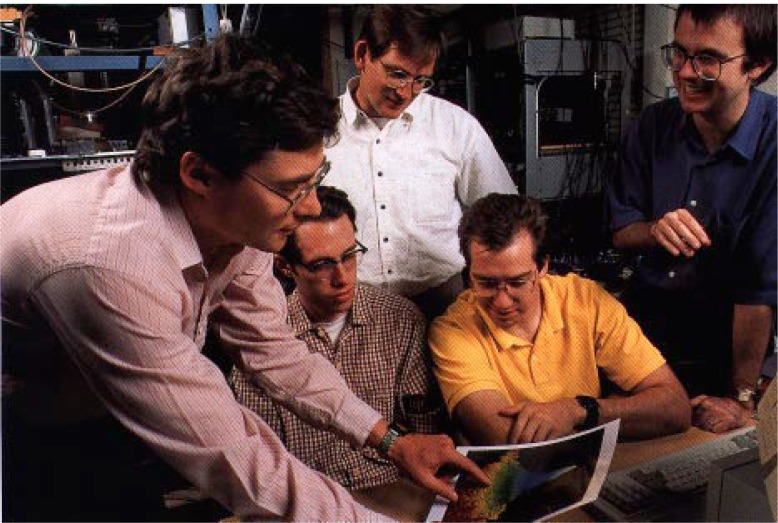
The JILA/University of Colorado team that first observed Bose-Einstein condensation in a gas. From left to right: Carl Wieman, Michael Matthews, Michael Anderson, Jason Ensher, and Eric Cornell. Their discovery was reported in the article, “Observation of Bose-Einstein Condensation in a Dilute Atomic Vapor,” by M. H. Anderson, J. R. Ensher, M. R. Matthews, C. E. Wieman, and E. A. Cornell, Science **269**, 198 (1995).

**Fig. 2:**
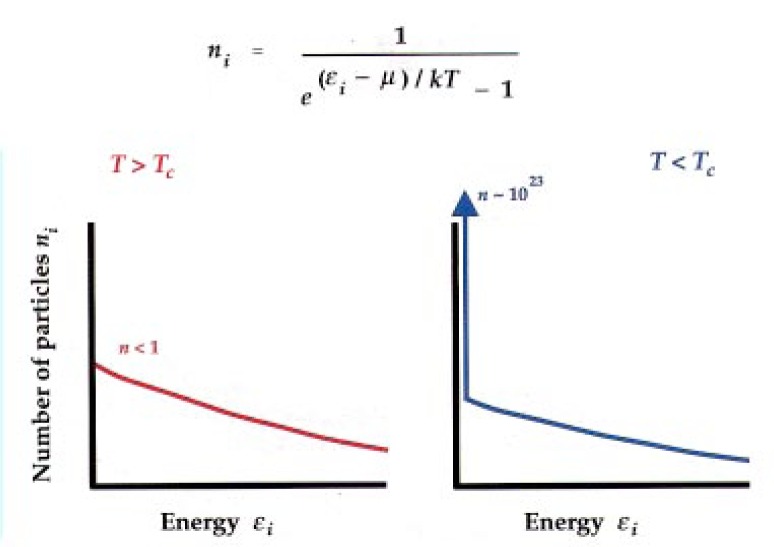
Schematic diagram of the Bose-Einstein distribution for a system of particles at a temperature *T*. The formula shows the average number of particles *n_i_* occupying a state *i* of energy *ϵ_i_*. The parameter *μ* is the chemical potential, which is the energy required to add an additional particle to the system. The left frame depicts the general behavior of this distribution above the transition temperature *T*_c_; the right panel shows the macroscopic occupancy of the lowest state of the system when *T* < *T*_c_.

**Fig. 3:**
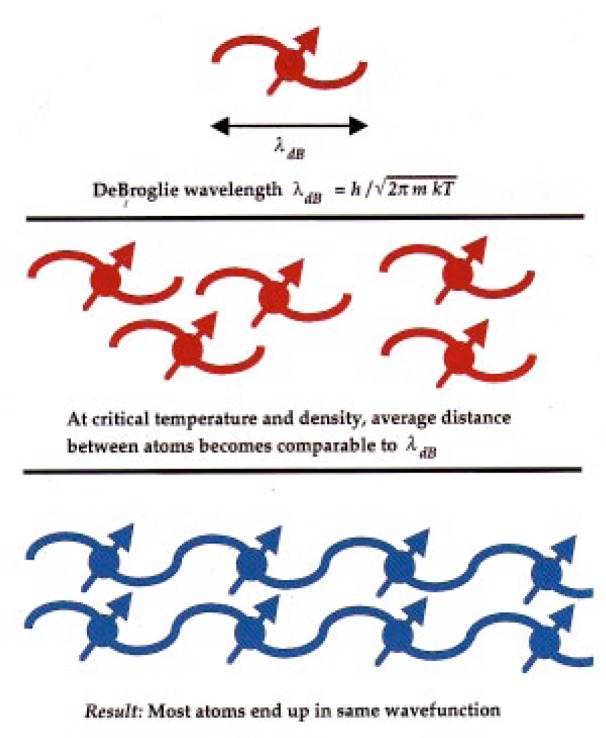
BEC occurs when the deBroglie wavelengths of the atoms in the gas become comparable to the average distance between gas atoms.

**Fig. 4:**
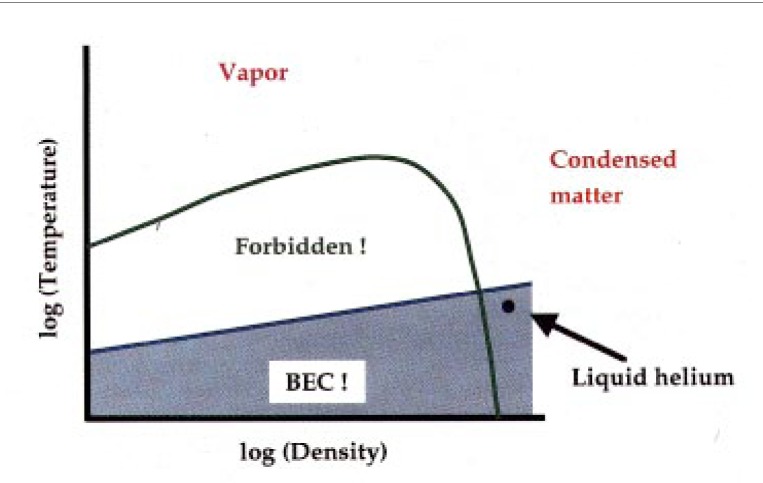
Schematic “universal” phase diagram of ordinary matter.

**Fig. 5:**
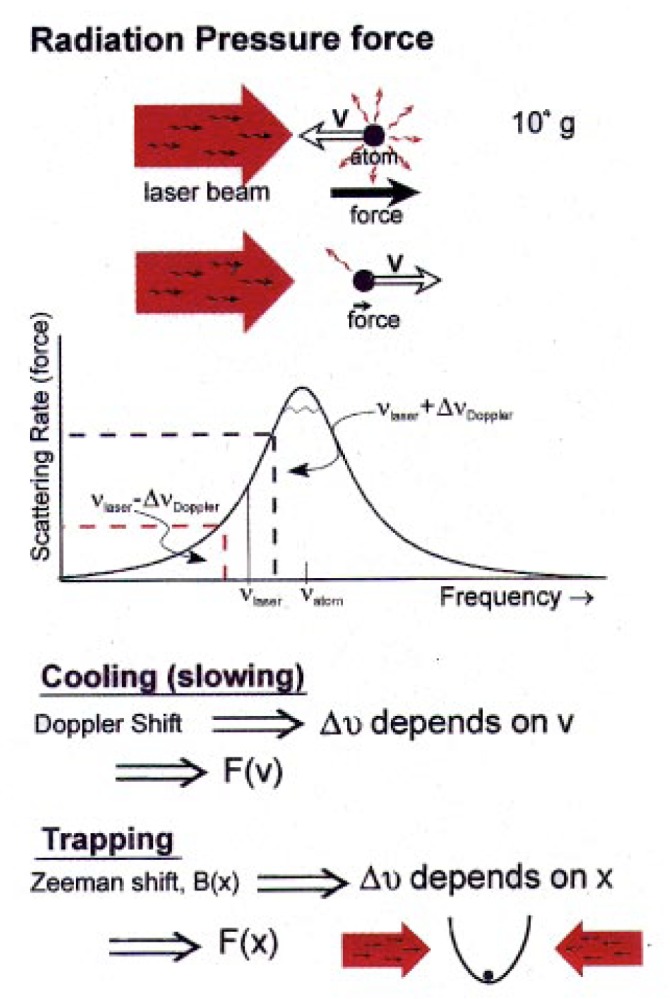
How laser cooling works.

**Fig. 6:**
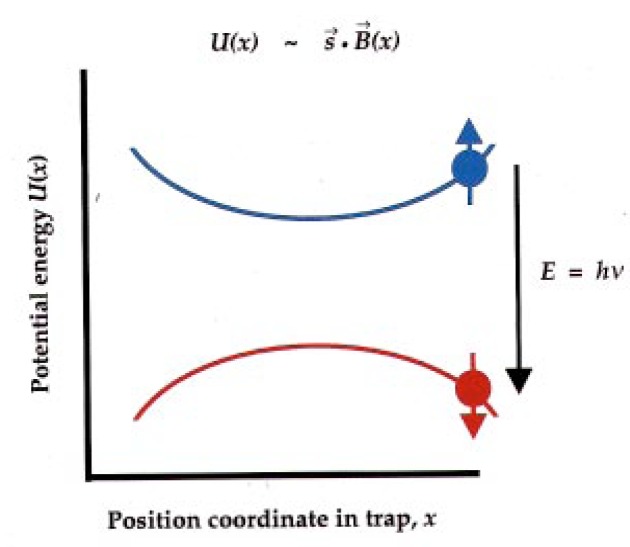
Principle of a magnetic trap for atoms. The magnetic field has a minimum value. An atom with spin parallel to the magnetic field (i.e., atomic magnetic moment antiparallel to the magnetic field), is attracted to the minimum; for spin antiparallel to the field, the atom is repelled from the minimum. A spin-flip can be induced by electromagnetic excitation at the resonance frequency *v* = *E*/*h* corresponding to the energy difference *E* between the two spin orientations, as shown (*h* = Planck’s constant). The resonance frequency lies in the radio portion of the electromagnetic spectrum; its precise value obviously depends upon the position in the trap.

**Fig. 7:**
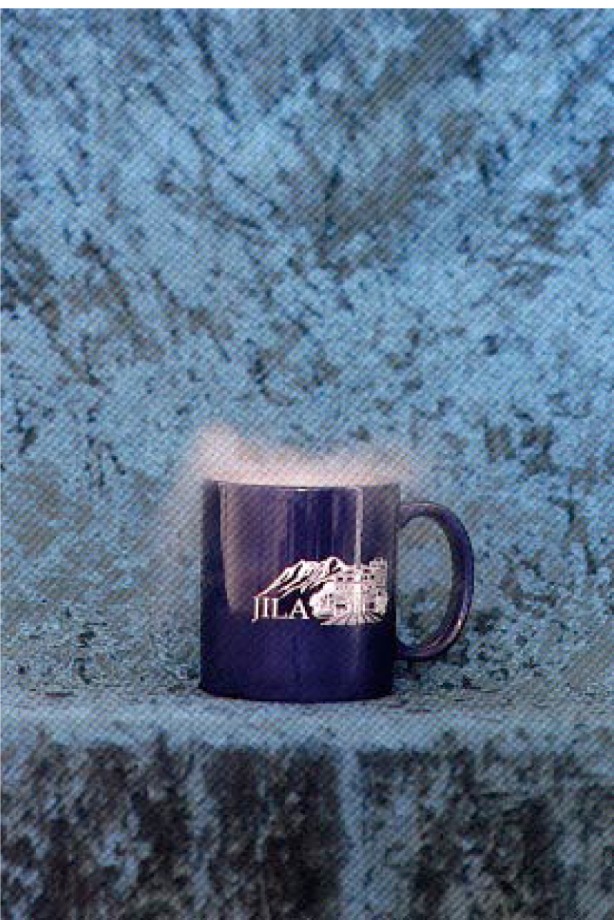
Evaporative cooling in action at JILA.

**Fig. 8:**
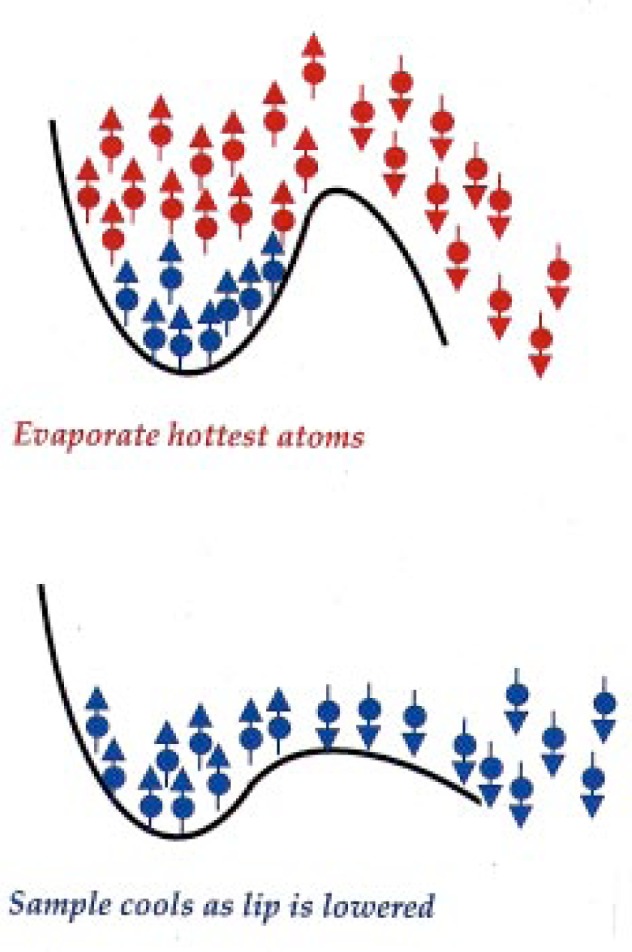
Radio-frequency evaporative cooling of trapped atoms. The resonance frequency (see Fig. 6) is chosen to expel the hottest atoms, which can be found highest in the bowl, and is gradually decreased to keep shaving off the hottest atoms in the sample.

**Fig. 9:**
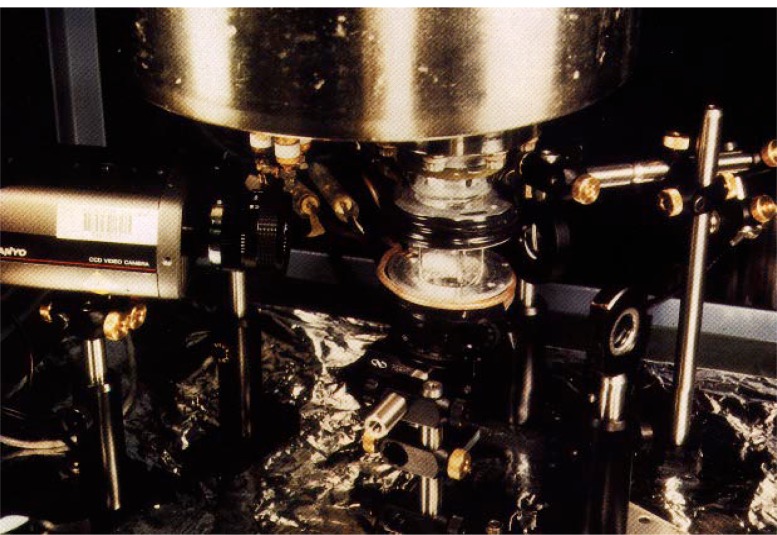
The experimental apparatus at JILA.

**Fig. 10:**
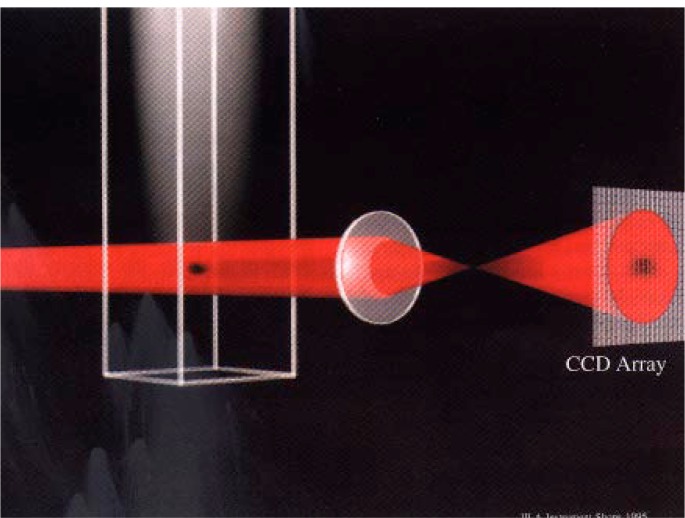
How atomic clouds are imaged.

**Fig. 11:**
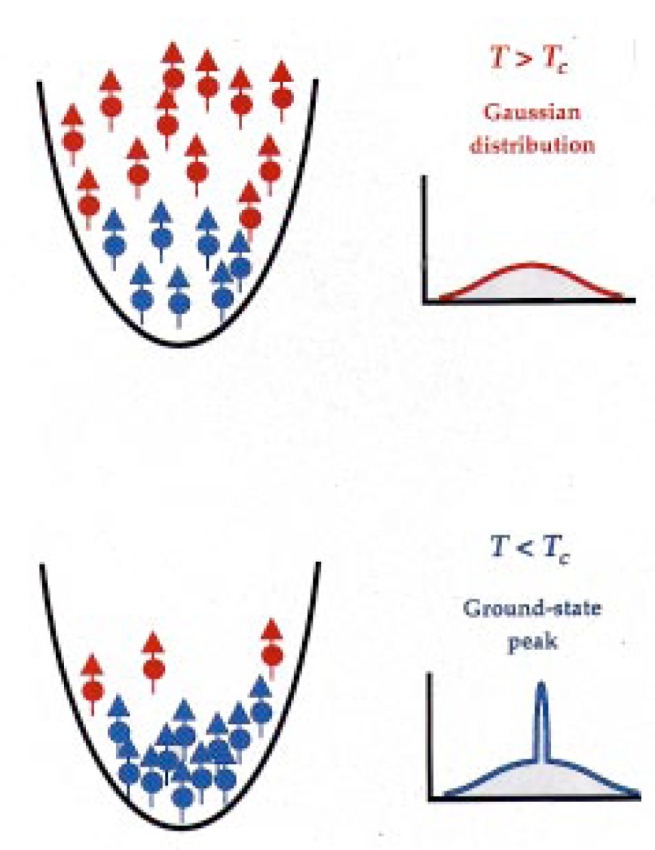
Spatial distribution of atoms in the expanded cloud, above and below the transition temperature *T*_c_. For *T* > *T*_c_, atoms are distributed among many energy levels of the system, and have a Gaussian distribution of velocities; for *T* < *T*_c_, the concentration of atoms in the lowest state gives rise to a pronounced peak in the distribution at low velocities.

**Fig. 12:**
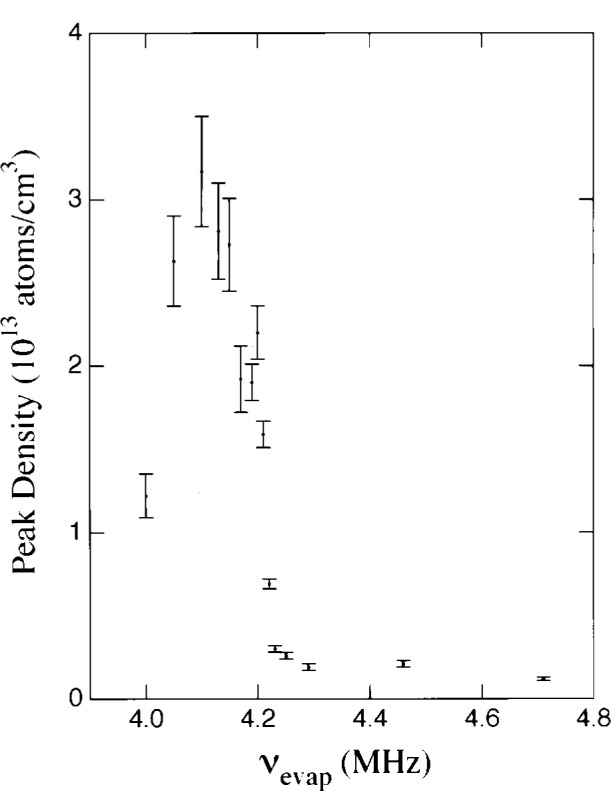
Atomic density as a function of RF evaporative cooling frequency.

**Fig. 13:**
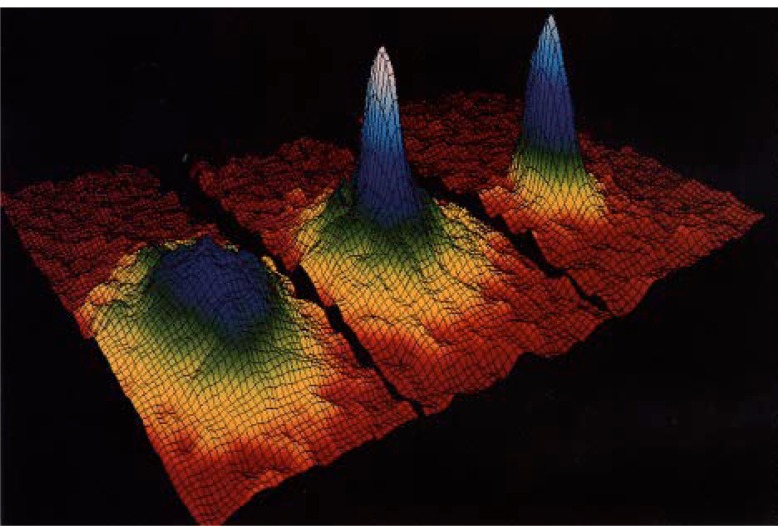
Images of the velocity distributions of the trapped atoms, taken by the expansion method. The left frame shows the velocity distribution just before the appearance of the Bose-Einstein condensate; the center frame, just after the appearance of the condensate; the right frame, after further evaporation leaves a sample of nearly pure condensate. The field of view of each frame is 200 μm × 270 μm, and corresponds to the distance the atoms have moved in about 1/20 s. The color corresponds to the number of atoms at each velocity, with red being the fewest and white being the most. Areas appearing white and light blue indicate lower velocities.

**Fig. 14:**
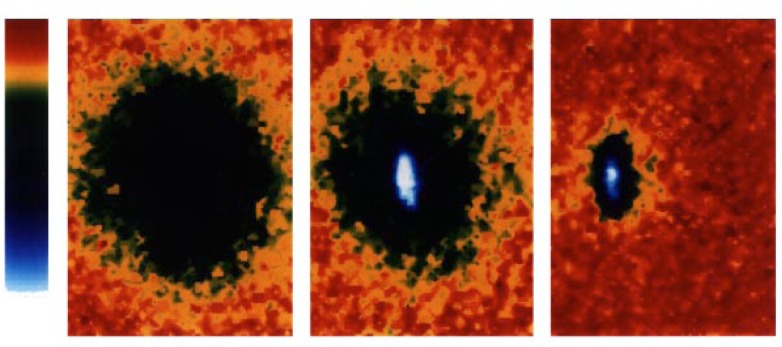
The data of Fig. 13 as seen from above, displayed in false color.

**Fig. 15:**
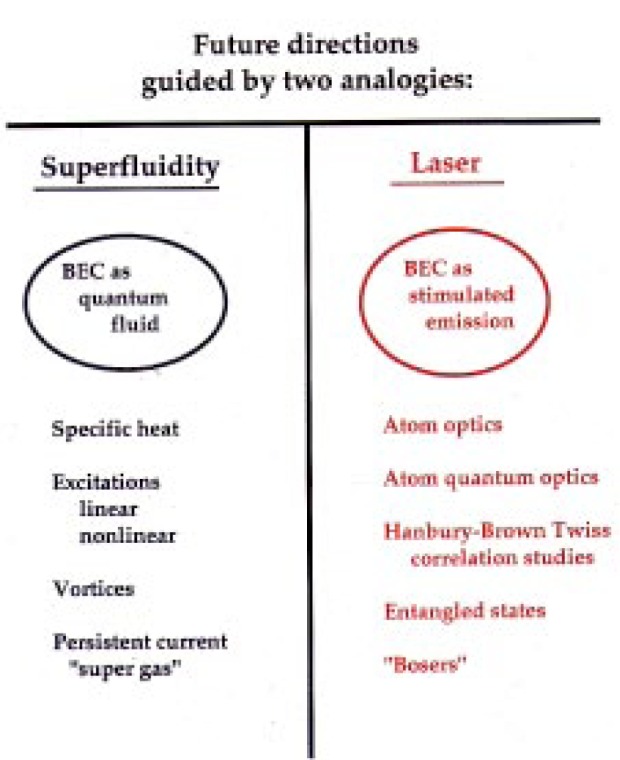
Future directions of BEC research.

